# Catalytic Asymmetric 1,4-Additions of β-Keto Esters to Nitroalkenes Promoted by a Bifunctional Homobimetallic Co_2_-Schiff Base Complex

**DOI:** 10.3390/molecules15010532

**Published:** 2010-01-22

**Authors:** Makoto Furutachi, Zhihua Chen, Shigeki Matsunaga, Masakatsu Shibasaki

**Affiliations:** Graduate School of Pharmaceutical Sciences, The University of Tokyo, 7-3-1 Hongo, Bunkyo-ku, Tokyo 113-0033, Japan

**Keywords:** asymmetric catalysis, asymmetric synthesis, bifunctional catalyst, Michael reaction, Schiff base

## Abstract

Catalytic asymmetric 1,4-addition of β-keto esters to nitroalkenes is described. 2.5 mol % of a homobimetallic Lewis acid/Brønsted base bifunctional Co_2_-Schiff base complex smoothly promoted the reaction in excellent yield (up to 99%), diastereoselectivity, and enantioselectivity (up to >30:1 dr and 98% ee). Catalyst loading was successfully reduced to 0.1 mol %. Mechanistic studies suggested that intramolecular cooperative functions of the two Co-metal centers are important for high catalytic activity and stereoselectivity.

## 1. Introduction

**Figure 1:**
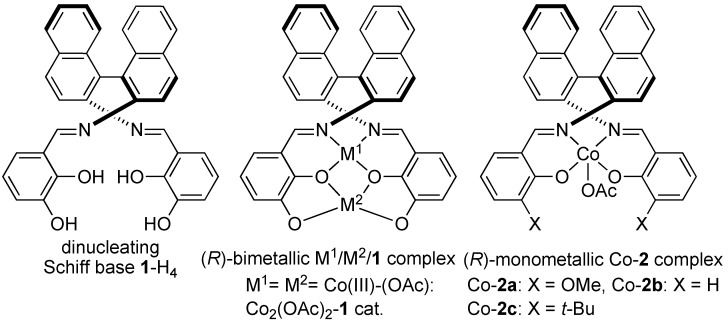
Structures of dinucleating Schiff base **1**-H_4_, bimetallic M^1^/M^2^ Schiff base complex and monometallic Co-salen **2a**–**2c **complexes .

Bifunctional concerto asymmetric catalysis is currently a hot research topic in organic synthesis. Various chiral bifunctional metal- and organo-catalysts have been reported over the last decade [1,2,3,4,5]. Bifunctional asymmetric catalysts are useful for realizing high stereoselectivity and catalytic activity *via* dual activation of both nucleophiles and electrophiles. As part of our ongoing research on this issue, we recently reported the utility of bimetallic Schiff base **1 **complexes (Figure 1), whose catalytic properties differ from those of well-established monometallic salen **2 **complexes [6,7,8]. By utilizing dinucleating Schiff bases, we developed heterobimetallic Cu/Sm [9], Pd/La [10,11], and Ga/Yb [12] Schiff base complexes, including rare earth metals and homobimetallic Ni_2 _[13,14,15,16,17,18], Co_2 _[19], and Mn_2 _[20] Schiff base **1 **complexes and applied them to various enantioselective reactions (for selected examples of related bifunctional bimetallic Schiff base catalysts, see ref [21,22,23,24,25,26,27]). In this manuscript, we report the details of our efforts to expand the utility of bimetallic Schiff base catalysis for catalytic asymmetric 1,4-addition of β-keto esters to nitroalkenes [28,29,30,31,32,33,34,35,36,37,38,39,40,41,42]. 

## 2. Results and Discussion

### 2.1. Homobimetallic Co_2_-Schiff Base Complex-catalyzed Asymmetric 1,4-Addition to Nitroalkenes

To find a suitable metal combination for the 1,4-addition reaction of β-keto esters to nitroalkenes, we selected nitroalkene **3a** and β-keto ester **4a** as model substrates for the construction of adjacent quaternary/tertiary carbon stereocenters [34,35,36,37,38,39,40,41,42]. The catalyst screening results are summarized in Table 1. Heterobimetallic Schiff base Cu/Sm and Pd-La complexes smoothly promoted the reaction, but neither diastereoselectivity nor enantioselectivity were satisfactory (entries 1–2). A homobimetallic Ni_2_-Schiff base **1 **complex (Figure 1) [13], which was suitable for 1,2-addition of β-keto esters to imines, gave moderate enantioselectivity (entry 3, 74% ee). Among other metals screened (entries 4–7), a Co_2_(OAc)_2_-**1** complex[19] gave product **5aa** in 95% ee (entry 4). Other homobimetallic Mn_2_-**1 **[20], Cu_2_-**1**, and Zn_2_-**1 **catalysts resulted in poor enantioselectivity (entry 5: 32% ee, entry 6: 12% ee, entry 7: 8% ee). Because the bimetallic Co_2_(OAc)_2_-**1** catalyst was stable against air and moisture, the reaction was successfully performed using undistilled THF (containing stabilizer and 220 ppm H_2_O) as a solvent under air atmosphere, and high enantioselectivity was achieved at room temperature with 2.5 mol % catalyst (entry 8). Notably, high yield and enantioselectivity were achieved even without solvent (entry 9, >99% conversion, 97% ee) under an air atmosphere.

**Table 1:** Screening of bimetallic M^1^**/**M^2^/Schiff base complexes for 1,4-addition of β-keto ester **4a **to nitroalkene **3a**.

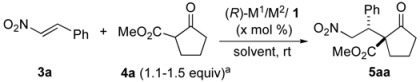								
Entry	M ^1^	M ^2^	cat. (mol %)	time (h)	solvent (M)	Dr ^b^	% yield ^b^	% ee
1	Cu	Sm-O*i*Pr	10	36	THF (0.4)	3:1	>99	23
2	Pd	La-O*i*Pr	10	36	THF (0.4)	4:1	>99	14 ^c^
3	Ni	Ni	10	36	THF (0.4)	6:1	71	74
4	Co-OAc	Co-OAc	10	6	THF (0.4)	26:1	86	95
5	Mn-OAc	Mn-OAc	10	36	THF (0.4)	3:1	61	32
6	Cu	Cu	10	36	THF (0.4)	9:1	6	12 ^c^
7	Zn	Zn	10	36	THF (0.4)	2:1	90	8
8 ^d^	Co-OAc	Co-OAc	2.5	9	THF (2.0)	18:1	94	93
9 ^e^	Co-OAc	Co-OAc	2.5	8	neat	25:1	>99	97

^a^ 1.5 equiv of **4a** was used in entries 1–7, and 1.1 equiv of **4a **in entries 8–9; ^b^ Yield and dr were determined by ^1^H-NMR analysis of crude mixtures; ^c^ *ent*-**4aa** was obtained in major; ^d^ Undistilled THF with stabilizer containing 220 ppm H_2_O was used; ^e^ Reaction was run under an open-air atmosphere.

The substrate scope is summarized in Table 2. Because the Co_2_(OAc)_2_-**1** catalyst is bench-stable and storable, the catalyst stored under air at room temperature for more than three months was used in Table 2 without loss of selectivity or reactivity. Furthermore, the reactions were performed under neat conditions at room temperature (24–28 °C) under an open-air atmosphere. In entries 1–9, the nitroalkene scope was investigated using β-keto ester **4a**. Nitroalkenes **3b**–**3d** with an electron-withdrawing substituent on the aromatic ring at the *para-* or *meta-*position reacted smoothly, giving products in 88%–94% yield, 30:1–>30:1 dr, and 97%–98% ee after 4–5 h (entries 2–4). The use of *ortho*-substituted nitroalkene **3e** slightly decreased the reactivity (77% yield, 14 h), but high diastereo- and enantioselectivity were maintained (entry 5, 27:1 dr, 94% ee). Nitroalkenes **3f**–**3g** with an electron-donating substituent on the aromatic ring as well as β-heteroaryl nitroalkene **3h** gave products in high yield and stereoselectivity (entries 6–8). The Co_2_(OAc)_2_-**1** catalyst also promoted the reaction of less reactive β-alkyl-nitroalkene **3i**, giving the product **5ia** in 96% yield, >30:1 dr, and 95% ee (entry 9). High diastereo- and enantioselectivity were also achieved with the six-membered ring β-keto ester **4b** (entry 10, >30:1 dr and 98% ee). The reaction rate, however, was decreased with β-keto ester **4b**, and the product **5bb** was obtained in 75% yield after 24 h. Acyclic β-keto ester **4c** also gave product **5ac **in high enantioselectivity (96% ee), but the reactivity and diastereoselectivity were decreased even with 10 mol % catalyst loading (entry 11, 73% yield, 3.3:1 dr). Trials to reduce catalyst loading are summarized in entries 12–13. Co_2_(OAc)_2_-**1** catalyst (0.2–0.1 mol %) promoted the reaction of nitroalekene **3b **with **4a** under highly concentrated conditions (THF, 20 M), while maintaining high enantioselectivity. In entry 12, pure **5ba **was isolated in 87% yield and 99% ee by recrystallization without column chromatography purification.

**Table 2:** Catalytic asymmetric 1,4-addition of β-keto esters to nitroalkenes using Co_2_(OAc)_2_-Schiff base **1** complex.^a^

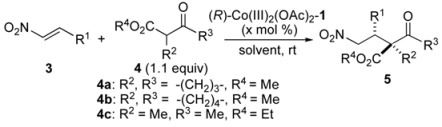										
Entry	R ^1^	3	4	cat. (x mol %)	time (h)	solvent (y M)	5	Dr ^b^	% yield^c^	% ee
1	Ph	**3a**	**4a**	2.5	8	neat	**5aa**	25:1	>99	97
2	4-Cl-C_6_H_4_	**3b**	**4a**	2.5	4	neat	**5ba**	>30:1	94	98
3	4-Br-C_6_H_4_	**3c**	**4a**	2.5	5	neat	**5ca**	>30:1	95	98
4	3-Br-C_6_H_4_	**3d**	**4a**	2.5	4	neat	**5da**	30:1	88	97
5	2-Br-C_6_H_4_	**3e**	**4a**	2.5	14	neat	**5ea**	27:1	77	94
6	4-MeO-C_6_H_4_	**3f**	**4a**	2.5	17	neat	**5fa**	9:1	93	94
7	4-Me-C_6_H_4_	**3g**	**4a**	2.5	10	neat	**5ga**	22:1	93	96
8	2-furyl	**3h**	**4a**	2.5	3	neat	**5ha**	>30:1	93	92
9	PhCH_2_CH_2_	**3i**	**4a**	2.5	7	neat	**5ia**	>30:1	96	95
10	4-Cl-C_6_H_4_	**3b**	**4b**	2.5	24	neat	**5bb**	>30:1	75	98
11^d^	Ph	**3a**	**4c**	10	36	neat	**5ac**	3.3:1	73	96
12	4-Cl-C_6_H_4_	**3b**	**4a**	0.2	24	THF (20)	**5ba**	>30:1	87^e^	99
13	4-Cl-C_6_H_4_	**3b**	**4a**	0.1	48	THF (20)	**5ba**	16:1	98	95

^a^ Reaction was performed under neat conditions at room temperature (24–28 °C) under air atmosphere with 1.1 equiv of **4** unless otherwise noted; ^b^ Dr was determined by ^1^H-NMR analysis; ^c^ Isolated yield after purification by column chromatography (entries 1–11 and 13); ^d^ 2.0 equiv of **4** was used; ^e^ **5ba** was obtained in pure form by recrystallization of the crude product without column chromatography purification.

### 2.2. Mechanistic Studies of Homobimetallic Co_2_(OAc)_2_-Schiff Base Complex

**Scheme 1:**
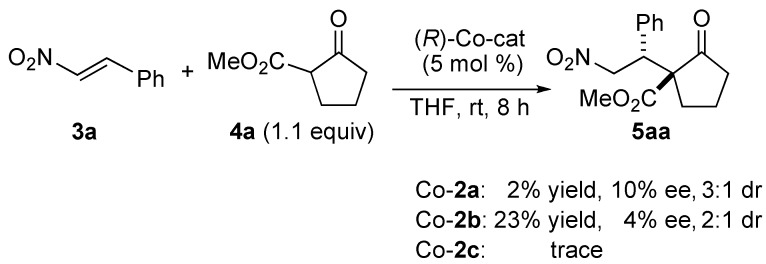
Negative control experiments using monomoetallic Co-salen **2a**–**2c **complexes.

**Figure 2:**
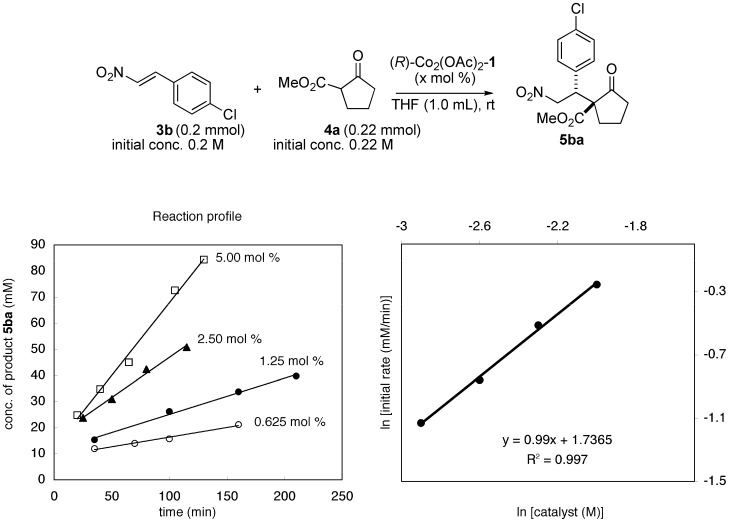
Initial rate kinetic studies of bimetallic Co_2_(OAc)_2_-**1 **catalyst.

**Figure 3:**
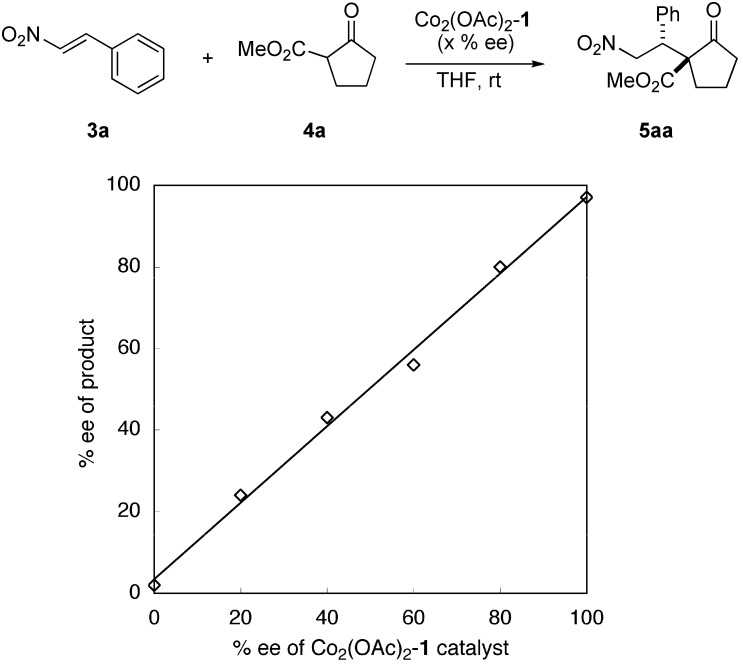
Linear relationship between% ee of bimetallic Co_2_(OAc)_2_-**1 **catalyst and% ee of product **5aa**.

**Scheme 2:**
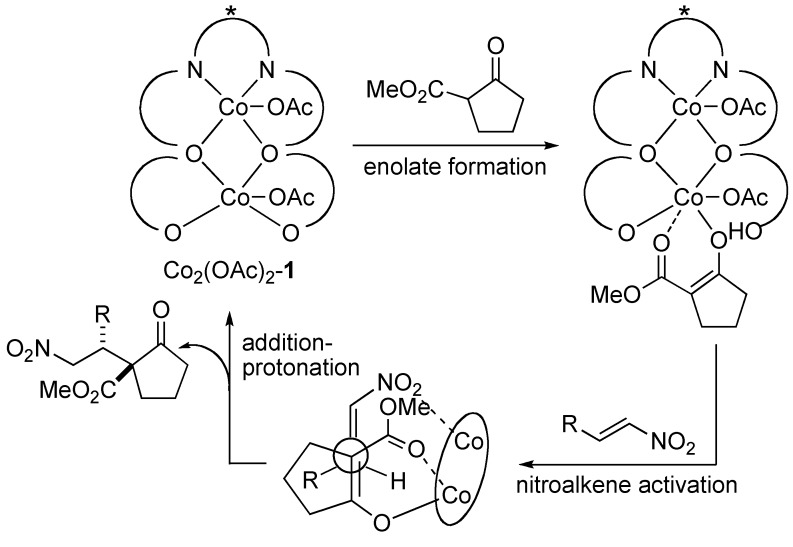
Postulated catalytic cycle of the reaction.

To gain mechanistic insight into the present homobimetallic Co_2_-catalysis, negative control experiments using three monometallic Co-salen **2a**–**2c** complexes with different substituents were investigated (Scheme 1). In all cases, poor yield, and poor diastereoselectivity and enantioselectivity were observed, suggesting that the bimetallic system is important for high catalytic activity as well as stereoselectivity. In addition, initial rate kinetic studies using nitroalkene **3b** and β-keto ester **4a** showed first-order dependency on the bimetallic Co_2_(OAc)_2_-Schiff base **1** complex (Figure 2). There was a linear relationship between the enantiomeric excess of the Co_2_(OAc)_2_-**1 **catalyst and product **5aa** (Figure 3). The results shown in Figure 2 and Figure 3 suggested that the active species in the present reaction would be a monomeric Co_2_(OAc)_2_-**1 **catalyst. Thus, the intramolecular concerto functions of the two Co metal centers are likely important in the present system, rather than intermolecular concerto function of the two catalysts, which was reported for mono-metallic Co-salen complexes [8]. The postulated catalytic cycle of the reaction is shown in Scheme 2. We assume that β-keto ester would coordinate to sterically less hindered outer Co-metal center of the Co_2_(OAc)_2_-**1 **catalyst. One of Co-aryloxide (or Co-acetate) would deprotonate α-proton of β-keto esters to generate Co-enolate. Inner Co-metal center would act as a Lewis acid to activate nitroalkenes in a similar manner as observed in the monomeric Co-salen system. 1,4-Addition via bimetallic transition state followed by protonation affords products and regenerates the Co_2_(OAc)_2_-**1 **catalyst.

## 3. Experimental

### 3.1. General

Infrared (IR) spectra were recorded on a JASCO FT/IR 410 Fourier transform infrared spectrophotometer. NMR spectra were recorded on JEOL JNM-LA500 spectrometer, operating at 500 MHz for ^1^H-NMR and 125.65 MHz for ^13^C-NMR. Chemical shifts in CDCl_3_ were reported in the δ scale relative to CHCl_3_ (7.24 ppm) for ^1^H-NMR. For ^13^C-NMR, chemical shifts were reported on the δ scale relative to CHCl_3_ (77.0 ppm) as an internal reference. Column chromatography was performed with silica gel Merck 60 (230–400 mesh ASTM). Optical rotations were measured on a JASCO P-1010 polarimeter. ESI mass spectra were measured on Waters micromass ZQ (for LRMS) and JEOL JMS-T100LC AccuTOF spectrometer (for HRMS). FAB mass spectra (for HRMS) were measured on a JEOL JMS-700 spectrometer. The enantiomeric excess (ee) was determined by HPLC analysis. HPLC was performed on JASCO HPLC systems consisting of the following: pump, PU-2080 plus; detector, UV-2075 plus, measured at 254 nm; column, DAICEL CHIRALCEL OD, CHIRALCEL OD-H, or CHIRALPAK AD-H; mobile phase, hexane/2-propanol. Anhydrous Co(OAc)_2_ was purchased from Aldrich and used as received.

### 3.2. Preparation of Co(III)_2_(OAc)_2_-Schiff Base 1 Complex

To a solution of (*R*)-Schiff base ligand **1**-H_4_ (1049 mg, 2.0 mmol) in EtOH (20 mL), was added Co(OAc)_2_ (708 mg, 4.0 mmol), and the mixture was stirred under air atmosphere for 12 h under reflux. After cooling down to room temperature, H_2_O (10 mL) was added to the mixture and the mixture was stirred for 1 h at room temperature under air atmosphere. The precipitate (Co_2_(OAc)_2_-**1** complex) was collected by filtration. Then, the solid was washed with H_2_O (×3), EtOH/hexane = 1:1 (×3), and Et_2_O. The solid was dried under reduced pressure to afford the Co_2_(OAc)_2_-Schiff base **1** complex (1.047 g, 66% yield) as a brown solid. The complex was used for the asymmetric reaction without further purification, and was stored under air at room temperature. Catalytic activity did not change for at least 6 months. Results in Tables 2 and 3 were collected using the Co_2_(OAc)_2_-**1** complex stored for over 3 months. The structure was assigned to be Co_2_(OAc)_2_-**1**•2H_2_O based on elemental analysis after recrystallization from THF/AcOEt. Anal. Calcd. for C_38_H_30_Co_2_N_2_O_10 _[Co_2_(OAc)_2_-**1**•2H_2_O]: C, 57.59; H, 3.82; N, 3.53; Found: C, 57.84; H, 3.76; N, 3.58.

### 3.3. General Procedure for Catalytic Asymmetric 1,4-Additions of β-Keto Esters to Nitroalkenes under Solvent-Free Conditions

To a vial were added Co_2_/Schiff base **1 **catalyst (7.92 mg, 0.01 mmol) and β-keto ester **4a **(55.3 µL, 0.44 mmol). After stirring the mixture for 5 min at room temperature, nitroalkene **3b **(73.4 mg, 0.4 mmol) was added at room temperature. The reaction mixture was stirred for 4 h at room temperature under air atmosphere, and the crude residue was analyzed by ^1^H-NMR to determine the diastereomeric ratio. The reaction mixture was purified by silica gel flash column chromatography (hexane/ethyl acetate = 3/1) to afford **5ba **(122.8 mg, 94% yield) as a colorless solid.

*(1S)-1-[(1R)-2-Nitro-1-phenylethyl]-2-oxo-cyclopentanecarboxylic Acid Methyl Ester* (**5aa**) [34,35,37]. **5aa** is a known compound. colorless oil; IR (neat) ν 2956, 1751, 1725, 1552, 1230, 1149 cm^−1^; ^1^H NMR (CDCl_3_) δ 1.76–2.06 (m, 4H), 2.30–2.40 (m, 2H), 3.74 (s, 3H), 4.06 (dd, *J* = 4.0, 11.0 Hz, 1H), 4.99 (dd, *J* = 11.0, 13.8 Hz, 1H), 5.14 (dd, *J* = 4.0, 13.8 Hz, 1H) , 7.20–7.32 (m, 5H); ^13^C NMR (CDCl_3_) δ 19.3, 31.0, 37.9, 46.1, 53.0, 62.4, 76.3, 128.3, 128.8, 129.2, 135.2, 169.7, 212.2; ESI-MS *m/z* 314 [M+Na]^+^; [α]_D_^21.0 ^+41.7 (*c* 0.844, CHCl_3_); HPLC (DAICEL CHIRALCEL OD, hexane/2-propanol = 90/10, flow 1.0 mL/min, detection at 220 nm) t_R_ 14.8 min (major) and 21.5 min (minor). Relative configuration of **5aa** was determined by comparing the ^1^H-NMR and ^13^C-NMR data with the reported data. Absolute configuration of **5aa** was determined by comparison of the sign of optical rotation with the reported data. Lit. [α]_D_^25^ + 36.5 (*c*, 0.84, CHCl_3_) [34,35].

*(1S)-1-[(1R)-1-(4-Chlorophenyl)-2-nitroethyl]-2-oxo-cyclopentanecarboxylic Acid Methyl Ester* (**5ba**) [37]. Colorless solid; IR (KBr) ν 2960, 1724, 1554, 1218 cm^−1^; ^1^H-NMR (CDCl_3_) δ 1.76–1.95 (m, 3H), 1.97–2.08 (m, 1H), 2.28–2.40 (m, 2H), 3.71 (s, 3H), 4.00 (dd, *J* = 4.0, 11.0 Hz, 1H), 4.93 (dd, *J* = 11.0, 13.7 Hz, 1H), 5.11 (dd, *J* = 4.0, 13.7 Hz, 1H), 7.15–7.20 (m, 2H), 7.22–7.27 (m, 2H); ^13^C NMR (CDCl_3_) δ 19.2, 31.2, 37.8, 45.5, 53.0, 62.1, 76.1, 128.9, 130.6, 133.8, 134.2, 169.6, 212.1; ESI-MS *m/z* 348, 350 [M+Na]^+^; HRMS calcd. for C_15_H_16_ClNO_5_Cs [M+Cs]^+^: 457.9771, found 457.9763; [α]_D_^25.6^ +42.6 (*c* 1.05, CHCl_3_); HPLC (DAICEL CHIRALCEL OD, hexane/2-propanol = 90/10, flow 1.0 mL/min, detection at 220 nm) t_R_ 20.3 min (major) and 34.5 min (minor).

*(1S)-1-[(1R)-1-(4-Bromophenyl)-2-nitroethyl]-2-oxo-cyclopentanecarboxylic Acid Methyl Ester* (**5ca**). Colorless solid; IR (KBr) ν 2958, 1756, 1720, 1552, 1232, 1155 cm^−1^; ^1^H-NMR (CDCl_3_) δ 1.77–1.99 (m, 3H), 2.00–2.12 (m, 1H), 2.31–2.42 (m, 2H), 3.73 (s, 3H), 4.00 (dd, *J* = 4.0, 11.5 Hz, 1H), 4.95 (dd, *J* = 11.5, 14.4 Hz, 1H), 5.12 (dd, *J* = 4.0, 13.4 Hz, 1H), 7.12–7.16 (m, 2H), 7.40–7.44 (m, 2H); ^13^C-NMR (CDCl_3_) δ 19.2, 31.1, 37.7, 45.5, 52.9, 62.0, 76.0, 122.3, 130.9, 131.8, 134.2, 169.5, 212.0; ESI-MS *m/z* 392, 394 [M+Na]^+^; HRMS calcd. for C_15_H_16_BrNO_5_Cs [M+Cs]^+^: 501.9266, found 501.9274; [α]_D_^25.6^ +40.2 (*c* 1.08, CHCl_3_); HPLC (DAICEL CHIRALCEL OD, hexane/2-propanol = 90/10, flow 1.0 mL/min, detection at 220 nm) t_R_ 25.0 min (major) and 37.6 min (minor).

*(1S)-1-[(1R)-1-(3-Bromophenyl)-2-nitroethyl]-2-oxo-cyclopentanecarboxylic Acid Methyl Ester* (**5da**). Colorless oil; IR (neat) ν 2922, 1751, 1726, 1554, 1230, 1147 cm^−1^; ^1^H-NMR (CDCl_3_) δ 1.82–1.98 (m, 3H), 2.08–2.17 (m, 1H), 2.31–2.44 (m, 2H), 3.74 (s, 3H), 3.94 (dd, *J* = 4.0, 11.0 Hz, 1H), 4.97 (dd, *J* = 11.0, 13.7 Hz, 1H), 5.17 (dd, *J* = 3.7, 13.7 Hz, 1H), 7.15–7.21 (m, 2H), 7.39–7.43 (m, 2H); ^13^C-NMR (CDCl_3_) δ 19.2, 31.5, 37.7, 45.7, 53.0, 62.0, 76.0, 122.7, 128.0, 130.2, 131.4, 132.2, 137.7, 169.6, 212.0; ESI-MS *m/z* 392, 394 [M+Na]^+^; HRMS calcd. for C_15_H_16_BrNO_5_Cs [M+Cs]^+^: 501.9266, found 501.9272; [α]_D_^25.6^ +13.1 (*c* 0.993, CHCl_3_); HPLC (DAICEL CHIRALCEL OD, hexane/2-propanol = 90/10, flow 1.0 mL/min, detection at 220 nm) t_R_ 21.7 min (major) and 27.2 min (minor).

*(1S)-1-[(1S)-1-(2-Bromophenyl)-2-nitroethyl]-2-oxo-cyclopentanecarboxylic Acid Methyl Ester* (**5ea**) [37]. Yellow solid; IR (KBr) ν 2960, 1724, 1554, 1242 cm^−1^; ^1^H-NMR (CDCl_3_) δ 1.85–1.98 (m, 2H), 2.04–2.15 (m, 1H), 2.16–2.24 (m, 1H), 2.44–2.50 (m, 2H), 3.72 (s, 3H), 4.48 (dd, *J* = 3.5, 10.7 Hz, 1H), 5.03 (dd, *J* = 10.7, 13.7 Hz, 1H), 5.45 (dd, *J* = 3.5, 13.7 Hz, 1H), 7.11 (ddd, *J* = 1.5, 7.5, 8.0 Hz, 1H), 7.28 (ddd, *J* = 1.2, 7.5, 8.0 Hz, 1H), 7.49 (dd, *J* = 1.5, 8.0 Hz, 1H), 7.55 (dd, *J* = 1.2, 8.0 Hz, 1H); ^13^C-NMR (CDCl_3_) δ 19.2, 32.8, 37.7, 43.7, 52.8, 62.0, 76.9, 126.6, 128.2, 128.8, 129.5, 133.4, 136.3, 169.8, 212.4; ESI-MS *m/z* 392, 394 [M+Na]^+^; HRMS calcd. for C_15_H_16_BrNO_5_Cs [M+Cs]^+^: 501.9266, found 501.9272; [α]_D_^25.6^ –22.9 (*c* 0.926, CHCl_3_); HPLC (DAICEL CHIRALCEL OD, hexane/2-propanol = 90/10, flow 1.0 mL/min, detection at 220 nm) t_R_ 14.8 min (major) and 21.3 min (minor).

(*1S)-1-[(1R)-1-(4-Methoxyphenyl)-2-nitroethyl]-2-oxo-cyclopentanecarboxylic Acid Methyl Ester* (**5fa**) [37]. Yellow solid; ^1^H-NMR (CDCl_3_) δ 1.75–2.07 (m, 4H), 2.27–2.43 (m, 2H), 3.73 (s, 3H), 3.75 (s, 3H), 4.03 (dd, *J* = 4.0, 11.0 Hz, 1H), 4.94 (dd, *J* = 11.0, 13.4 Hz, 1H), 5.08 (dd, *J* = 4.0, 13.4 Hz, 1H), 6.78–6.83 (m, 2H), 7.11–7.16 (m, 2H); ^13^C-NMR (CDCl_3_) δ 19.2, 30.7, 37.9, 45.4, 52.9, 55.0, 62.5, 76.4, 114.0, 126.8, 130.3, 159.2, 169.8, 212.3; ESI-MS *m/z* 344 [M+Na]^+^; HRMS calcd. for C_16_H_19_NO_6_Cs [M+Cs]^+^: 454.0267, found 454.0271; [α]_D_^26.4^ +38.8 (*c* 0.210, CHCl_3_); HPLC (DAICEL CHIRALCEL OD, hexane/2-propanol = 90/10, flow 1.0 mL/min, detection at 220 nm) t_R_ 22.8 min (major) and 28.5 min (minor).

*(1S)-1-[(1R)-1-(4-Methylphenyl)-2-nitroethyl]-2-oxo-cyclopentanecarboxylic Acid Methyl Ester* (**5ga**) [37]. Colorless oil; IR (neat) ν 2956, 1751, 1725, 1554, 1230, 1149 cm^−1^; ^1^H-NMR (CDCl_3_) δ 1.75–2.06 (m, 4H) , 2.28 (s, 3H), 2.28–2.40 (m, 2H), 3.74 (s, 3H), 4.03 (dd, *J* = 4.0, 11.0 Hz, 1H), 4.96 (dd, *J* = 11.0, 13.8 Hz, 1H), 5.11 (dd, *J* = 4.0, 13.8 Hz, 1H) , 7.02–7.12 (m, 4H); ^13^C-NMR (CDCl_3_) δ 19.2, 20.9, 30.8, 37.9, 45.7, 52.9, 62.4, 76.3, 129.0, 129.4, 131.9, 137.9, 169.7, 212.2; ESI-MS *m/z* 328 [M+Na]^+^; HRMS calcd. for C_16_H_19_NO_5_Cs [M+Cs]^+^: 438.0318, found 438.0329; [α]_D_^26.0^ +24.0 (*c* 1.04, CHCl_3_); HPLC (DAICEL CHIRALCEL OD, hexane/2-propanol = 90/10, flow 1.0 mL/min, detection at 220 nm) t_R_ 14.0 min (major) and 18.4 min (minor).

*(1S)-1-[(1R)-1-Furan-2-yl-2-nitroethyl]-2-oxo-cyclopentanecarboxylic Acid Methyl Ester* (**5ha**). Yellow solid; IR (KBr) ν 2962, 1752, 1718, 1554, 1238, 1145, cm^−1^; ^1^H-NMR (CDCl_3_) δ 1.66–1.77 (m, 1H), 1.89–2.01 (m, 2H), 2.06–2.14 (m, 1H), 2.28–2.38 (m, 1H), 2.42–2.49 (m, 1H), 3.73 (s, 3H), 4.40 (dd, *J* = 4.3, 10.0 Hz, 1H), 4.87 (dd, *J* = 10.0, 13.4 Hz, 1H), 4.91 (dd, *J* = 4.3, 13.4 Hz, 1H), 6.16 (brd, *J* = 3.4 Hz, 1H), 6.25–6.29 (dd, *J* = 1.9, 3.4 Hz, 1H), 7.29–7.32 (m, 1H); ^13^C-NMR (CDCl_3_) δ 19.3, 30.0, 37.8, 40.3, 53.0, 61.7, 74.3, 109.9, 110.7, 142.6, 148.9, 169.3, 211.9; ESI-MS *m/z* 304 [M+Na]^+^; HRMS calcd. for C_1__3_H_15_NNaO_6_ [M+Na]^+^: 304.0797, found 304.0787; [α]_D_^25.6^ +64.4 (*c* 1.03, CHCl_3_); HPLC (DAICEL CHIRALCEL OD, hexane/2-propanol = 90/10, flow 1.0 mL/min, detection at 220 nm) t_R_ 12.8 min (major) and 20.0 min (minor). 

*(1S)-1-[(2R)-1-Nitro-4-phenylbutan-2-yl]-2-oxo-cyclopentanecarboxylic Acid Methyl Ester* (**5ia**). Colorless solid; IR (KBr) ν 2951, 2927, 1734, 1711, 1545, 1232 cm^−1^; ^1^H-NMR (CDCl_3_) δ 1.57–1.67 (m, 1H), 1.74–2.02 (m, 4H), 2.22–2.32 (m, 1H), 2.36–2.44 (m, 1H), 2.51–2.59 (m, 2H), 2.68–2.77 (m, 1H), 2.81–2.88 (m, 1H), 3.68 (s, 3H), 4.44 (dd, *J* = 5.5, 14.0 Hz, 1H), 4.95 (dd, *J* = 5.2, 14.0 Hz, 1H), 7.11–7.15 (m, 2H), 7.17–7.23 (m, 1H), 7.24–7.31 (m, 2H); ^13^C-NMR (CDCl_3_) δ 19.2, 31.2, 32.4, 33.9, 38.0, 39.9, 52.7, 62.7, 76.2, 126.3, 128.3, 128.5, 140.5, 169.8, 213.1; ESI-MS *m/z* 342 [M+Na]^+^; HRMS calcd. for C_1__7_H_21_NNaO_5_ [M+Na]^+^: 342.1317, found 342.1325; [α]_D_^25.5^ +76.2 (*c* 1.08, CHCl_3_); HPLC (DAICEL CHIRALCEL AD-H, hexane/2-propanol = 4/1, flow 1.0 mL/min, detection at 220 nm) t_R_ 7.3 min (major) and 7.9 min (minor). 

*(1S)-1-[(1R)-1-(4-Chlorophenyl)-2-nitroethyl]-2-oxo-cyclohexanecarboxylic Acid Methyl Ester* (**5bb**). Colorless oil; IR (neat) ν 2951, 1712, 1554, 1492, 1236 cm^−1^; ^1^H-NMR (CDCl_3_) δ 1.44–1.52 (m, 1H), 1.54–1.78 (m, 3H), 1.99–2.06 (m, 1H), 2.09–2.16 (m, 1H), 2.41–2.55 (m, 2H), 3.73 (s, 3H), 3.98 (dd, *J* = 3.4, 11.3 Hz, 1H), 4.74 (dd, *J* = 11.3, 13.4 Hz, 1H), 5.01 (dd, *J* = 3.4, 13.4 Hz, 1H), 7.09–7.12 (m, 2H), 7.24–7.29 (m, 2H); ^13^C-NMR (CDCl_3_) δ 22.3, 27.7, 36.9, 41.3, 47.2, 52.6, 63.0, 77.3, 126.7, 130.7, 133.9, 134.2, 170.0, 206.7; ESI-MS *m/z* 362, 364 [M+Na]^+^; HRMS calcd. for C_16_H_18_ClNO_5_Cs [M+Cs]^+^: 471.9928, found 471.9925; [α]_D_^26.5^ –73.0 (*c* 1.03, CHCl_3_); HPLC (DAICEL CHIRALCEL OD-H, hexane/2-propanol = 90/10, flow 1.0 mL/min, detection at 220 nm) t_R_ 13.4 min (major) and 8.9 min (minor).

*(2S,3R)-Ethyl 2-acetyl-2-methyl-4-nitro-3-phenylbutanoate* (**5ac**). Colorless oil; IR (neat) ν 2923, 1734, 1710, 1552, 1093, 701 cm^−1^; ^1^H-NMR (CDCl_3_) δ 1.21 (s, 3H), 1.29 (t, *J* = 7.3Hz, 3H), 2.15 (s, 3H), 4.12 (dd, *J* = 3.4, 11 Hz, 1H), 4.26 (q, *J* = 7.3 Hz, 2H), 4.87 (dd, *J* = 3.4, 13.4 Hz, 1H), 4.94 (dd, *J* = 11, 13.4 Hz, 1H), 7.08–7.15 (m, 2H), 7.25–7.31 (m,3H); ^13^C-NMR (CDCl_3_) δ 14.0, 20.1, 26.5, 47.7, 62.1, 62.5, 77.5, 128.4, 128.8, 129.0, 135.4, 171.2, 204.2; ESI-MS *m/z* 316 [M+Na]^+^; HRMS calcd. for C_1__5_H_19_NNaO_5_ [M+Na]^+^: 316.1161, found 316.1156; [α]_D_^25.0^ –53.1 (*c* 0.20, CHCl_3_); HPLC (DAICEL CHIRALCEL OD, hexane/2-propanol = 40/10, flow 1.0 mL/min, detection at 220 nm) t_R_ 7.1 min (major) and 19.2 min (minor).

## 4. Conclusions

In summary, we developed a highly enantioselective catalytic asymmetric 1,4-addition of β-keto esters to nitroalkenes for the construction of adjacent quaternary/tertiary carbon stereocenters. Bifunctional Co_2_-Schiff base **1 **complex smoothly promoted the reaction in excellent yield (up to 99%), diastereoselectivity, and enantioselectivity (up to >30:1 dr and 98% ee). Catalyst loading was successfully reduced to 0.1 mol %. Mechanistic studies suggested that intramolecular cooperative functions of the two Co-metal centers are important for high catalytic activity and stereoselectivity.
